# Laparoscopic repair of Morgagni hernia by primary closure with extra-abdominal suture: a case report and review of the literature

**DOI:** 10.11604/pamj.2024.47.150.43103

**Published:** 2024-03-28

**Authors:** Makoto Hasegawa, Yohei Sanmoto, Shunji Kinuta

**Affiliations:** 1 Department of Surgery, Takeda General Hospital, Yamagachou, Aizuwakamatsu-shi, Fukushima, Japan

**Keywords:** Extra-abdominal suture, laparoscopic repair, Morgagni hernia, primary closure, case report

## Abstract

We report a case of a Morgagni hernia repaired by primary closure with an extra-abdominal suture. Moreover, we reviewed cases of laparoscopically repaired Morgagni hernia, in which the size of the hernia defect was known, to establish a size criterion for mesh utilization. An 87-year-old woman presented to our hospital with right upper abdominal pain and vomiting. She had no history of abdominal surgery or trauma. Chest radiography and computed tomography (CT) revealed a Morgagni hernia, with the stomach and transverse colon herniated into the right chest cavity. Initially, an endoscopic repair was performed for the herniated stomach due to her age, which was successful. However, she had a recurrence 2 days later, prompting us to perform a semi-emergent laparoscopic surgery. Laparoscopic examination revealed a Morgagni defect, with the omentum, transverse colon, and stomach herniated, with the stomach reduced by pneumoperitoneum. Fortunately, the herniated organs could be easily relocated into the abdomen with no adhesions. The hernia defect measured 6 x 3 cm. We performed primary closure with an extra-abdominal suture. No sac resection was performed. The operation lasted 98 min. Oral intake was initiated on postoperative day 1, and the patient was discharged on postoperative day 3 without complications. Chest radiography and CT scans at 1 month postoperatively showed no recurrence, and the patient remained asymptomatic at the 9-month follow-up examination. According to our review findings, primary closure is an efficient method for small hernia defects (rule of thumb: width, <4 cm; length, <7 cm).

## Introduction

Morgagni hernia is a congenital defect in the anterior diaphragm, between its costal and sternal portions [1-4]. Regardless of symptoms, surgical intervention is advised in healthy individuals to avoid potential complications, such as obstruction and strangulation [1,2]. Recently, laparoscopic repair has become the preferred approach due to its safety, low morbidity rates, and short hospital stays [1-5]. However, the criteria for mesh repair remain unclear.

In this report, we present a case of a Morgagni hernia repaired laparoscopically by primary closure with an extra-abdominal suture. In addition, cases of laparoscopically repaired Morgagni hernia, in which the size of the hernia defect was known, were reviewed to establish a size criterion for mesh utilization.

## Patient and observation

**Patient information:** an 87-year-old woman presented to our hospital.

**Clinical findings:** the patient presented with right upper abdominal pain and vomiting.

**Timeline of current episode:** her medical history included coronary artery bypass grafting, with no utilization of abdominal vessels for grafting, and no recorded history of abdominal surgery or trauma.

**Diagnostic assessment:** chest radiography and computed tomography (CT) were performed.

**Figure 1:**
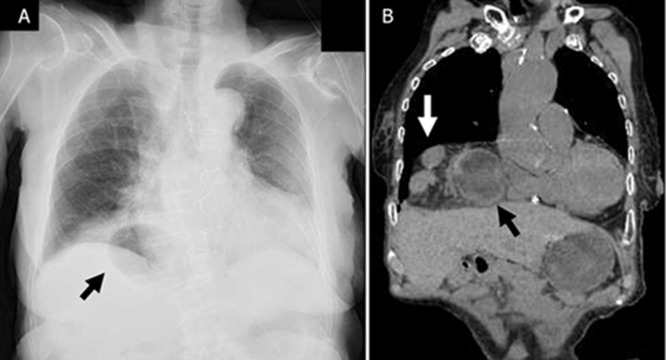
A) preoperative chest radiograph: gastrointestinal gas is observed at the diaphragm (black arrow); B) preoperative computed tomography. Herniation of the stomach (black arrow) and colon (white arrow) is observed

**Diagnosis:** our findings revealed a Morgagni hernia, with the stomach and transverse colon herniated into the right chest cavity (Figure 1 A, B). The initial strategy was to attempt endoscopic repair and then proceed to elective surgery.

Although the endoscopic repair was initially successful, unfortunately, the hernia recurred 2 days later, accompanied by persistent vomiting and difficulty in resuming oral intake. As a result, semi-emergency surgery was performed 3 days after the recurrence of the Morgagni hernia. The first step was to perform the surgery laparoscopically, and in the event of intraoperative pneumoperitoneum causing instability in vital signs, a decision was made to switch to open surgery.

**Figure 2:**
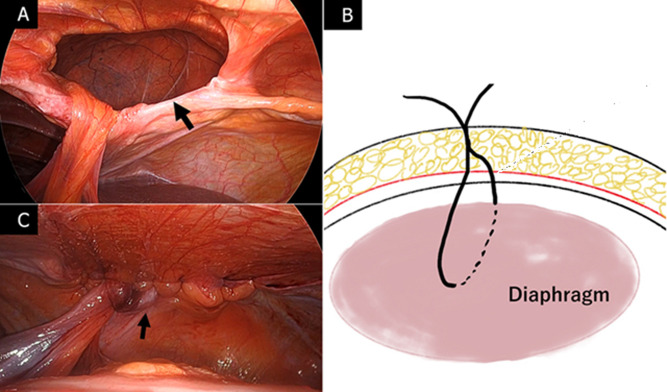
A) laparoscopic views of the defect (black arrow): the defect was 6 x 3 cm in size; B) schema of extra-abdominal suture (through the same skin incision 1 cm caudal to the previous fascial opening); C) laparoscopic views after primary closure, tension-free repair of the hernia defect is achieved

**Therapeutic interventions:** the patient was placed in an open-leg reverse trendelenburg position. The first 12-mm trocar was placed through the umbilicus using an open technique. Additional 12-mm and 5-mm trocars were placed under laparoscopic guidance. The pneumoperitoneal pressure was maintained at 10 mmHg. Laparoscopic examination revealed a Morgagni defect; the omentum, transverse colon, and stomach were herniated, with the stomach reduced by pneumoperitoneum. Fortunately, the herniated organs could be easily relocated into the abdomen with no adhesions. The hernia defect measured 6 x 3 cm (Figure 2A). To ensure tension-free closure, pneumoperitoneal pressure was reduced to 4 mmHg, and primary closure was performed. Skin incisions measuring 2 mm were made, through which 0 SURGILON® (Medtronic, Dublin, Ireland) sutures were passed into the abdomen using Lapa-Her-Closure® (Hakko, Chikuma City, Japan). Each suture was laparoscopically inserted through the fascia to the posterior rim.

Subsequently, the needle end was retrieved with Lapa-Her-Closure® through the same skin incision, positioned 1 cm caudal to the previous fascial opening, ensuring a secure tie to the fascia (Figure 2B). All eight sutures were placed; the tension was evenly distributed by gently pulling up the sutures, after which each suture was tied (Figure 2C), and the knots were placed in the fascia supra. No sac resection was performed. The operation lasted 98 min. Oral intake was initiated on postoperative day 1, and the patient was discharged on postoperative day 3, with no complications.

**Figure 3:**
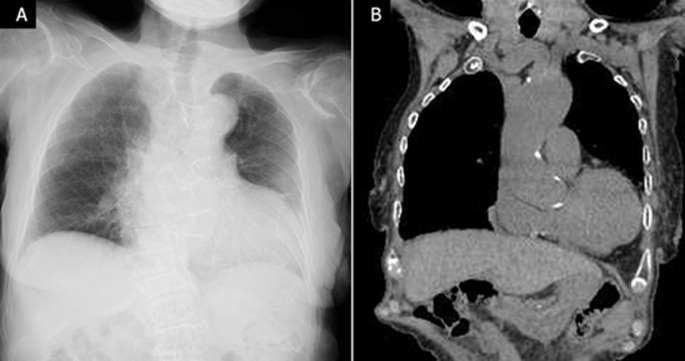
A) chest radiograph at 1-month follow-up examination (no recurrence is observed); B) computed tomography at 1-month follow-up examination (no recurrence is observed)

**Follow-up and outcome of interventions:** chest radiography and computed tomography (CT) findings at 1 month postoperatively showed no recurrence (Figure 3 A, B), and the patient remained asymptomatic at the 9-month follow-up examination.

**Patient perspective:** the patient and her family were satisfied with our treatment and follow-up. They thanked all the medical staff involved in her treatment.

**Informed consent:** written informed consent was obtained from the patient for publication of this case report.

## Discussion

The use of mesh is generally recommended for large defects [1,6,7]; however, there is no clear cutoff value for Morgagni defect size. In our case, the defect was small (6 x 3 cm) and, therefore, we performed primary closure and had a good postoperative course. To establish a size criterion for mesh utilization, we conducted a comprehensive literature review of laparoscopically repaired Morgagni hernia, in which the size of the hernia defect was known.

**Figure 4:**
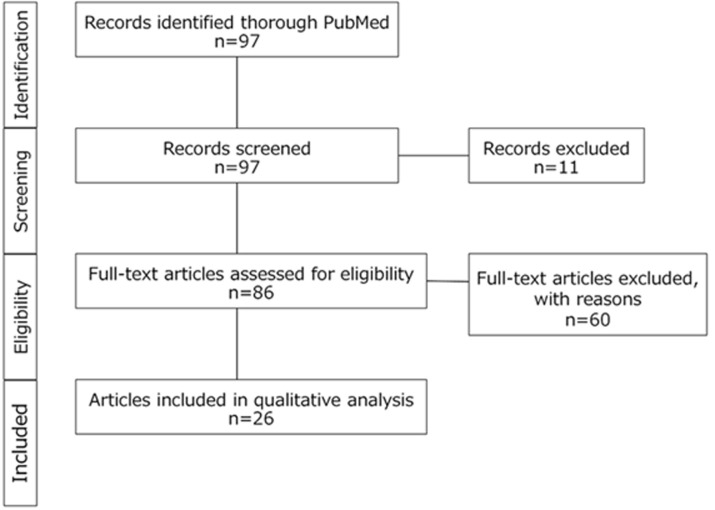
flow diagram demonstrating study selection for meta-analysis

The search conducted using PubMed employed keywords such as “Morgagni hernia”, “laparoscopic repair”, and “adult”. The search yielded 97 articles, 11 of which were excluded because they did not report laparoscopic repair of Morgagni hernia. Furthermore, 60 articles were excluded because they lacked a description of the hernial defect size. The remaining 26 articles (comprising 35 cases) in addition to our present case, were included in the quantitative analysis, totaling 36 cases (Figure 4). Notably, none of the cases in the analysis experienced a recurrence. Statistical analyses, including t-tests and Fisher´s exact tests, were performed to confirm the association between primary closure and mesh repair groups, considering factors, such as age, sex, hernia size, and types of herniated organs. To determine the cutoff values for defect size, we performed a receiver operating characteristic (ROC) curve analysis. All statistical analyses were performed using EZR (Saitama Medical Center, Jichi Medical University, Saitama, Japan), which is a graphical user interface for R (The R Foundation for Statistical Computing, Vienna, Austria). More precisely, it is a modified version of R commander designed to add statistical functions frequently used in biostatistics [8].

**Figure 5:**
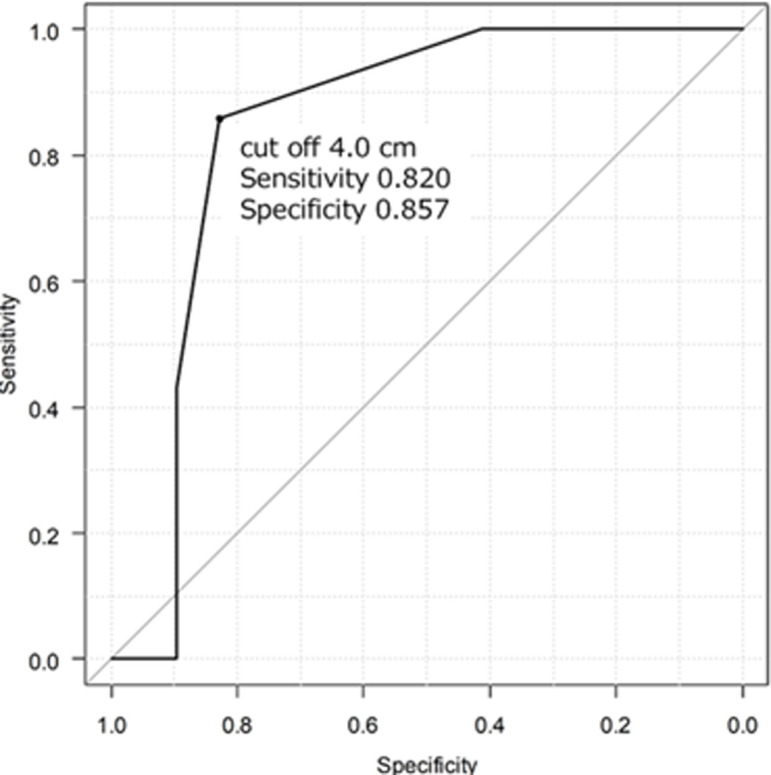
receiver operating characteristic curve between the Morgagni defect width and use of mesh

**Figure 6:**
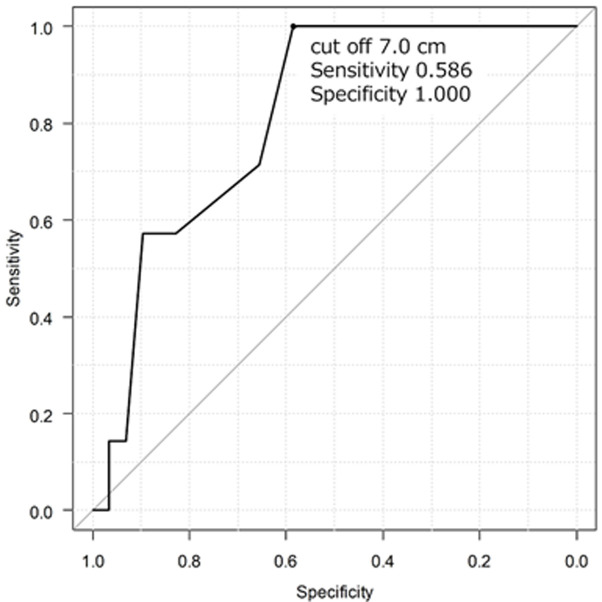
receiver operating characteristic curve between the Morgagni defect length and use of mesh

Out of the total cases analyzed, primary closure was employed in seven cases, while mesh repair was conducted in 29 cases, with no reported cases of post-operative recurrence. The majority of cases involved female patients, comprising 11 (30.6%) male patients. The mean age at presentation was 62.7 years. The mean width of the Morgagni defect was 5.3 cm, and the mean length of the Morgagni defect was 7.4 cm. The most frequently herniated organ was the colon (27 cases), followed by the omentum, stomach, and small bowel (Table 1). According to the receiver operating characteristic (ROC) curve analysis, defects ≥4.0 cm width (area under the curve, 0.842; sensitivity, 0.828; specificity, 0.857) and ≥7.0 cm length (area under the curve, 0.813; sensitivity, 0.586; specificity, 1.000), were more likely to be repaired with mesh (Figure 5, Figure 6).

**Table 1:** demographic data of patients included in our literature reviewed patients

	All patients (n=36)
Male	11(30.6%)
Age at operation time, mean (SD) years	62.7±17.4
Width of Morgagni defect, mean (SD) cm	5.3±2.1
Length of Morgagni defect, mean (SD) cm	7.4±3.0
Herniated organ: omentum	26(72.2%)
Herniated organ: colon	27(75%)
Herniated organ: stomach	8(22.2%)
Herniated organ: small bowel	3(8.3%)
Recurrence	0(0%)
Primary closure	7(19.4%)

SD: standard deviation

This case study and meta-analysis provide an indication of the size of defects for which mesh repair should be used (rule of thumb: width, <4 cm; length, <7 cm). For overall diaphragmatic hernia, it is reported that defects >20-30 cm^2^ should be repaired using mesh [6]. This value was consistent with the results of our study.

The opinion is divided on whether or not to perform sac resection for Morgagni hernia. While some recommend resection, others argue that sac resection should not be performed due to the possibility of serious complications, such as pneumopericardium [1]. In our case, sac resection was not performed because of the patient's age and concerns regarding possible complications.

We suggest reducing the pneumoperitoneal pressure, ensuring tension-free defect closure, and incorporating mesh reinforcement in cases where tension is a concern. A previous study reported that mesh was added after primary closure if there was any tension in the primary closure [7].

If primary closure is selected, the use of intracorporeal or extracorporeal ligation remains controversial. The absence of the anterior rim of the Morgagni defect makes intracorporeal suturing challenging. This difficulty can be avoided with extra-abdominal suturing. Furthermore, extra-abdominal suturing involves the entire abdominal wall and strengthens the reinforcement [9], with secure ligatures achievable by equalizing tension and tying each suture [10]. In a previous study, the pneumoperitoneal pressure was reduced prior to ligation to facilitate tension-free repair, as demonstrated here [10]. Our literature review had some limitations. First, the sample size was small and may not have been representative of the entire Morgagni hernia patient population. Second, publication bias may be possible, and cases with postoperative recurrence may have been excluded from our literature review.

## Conclusion

We report a case of Morgagni hernia repaired laparoscopically by primary closure. According to our case and review of the literature, primary closure is an effective method for small hernia defects (rule of thumb: width, <4 cm; length, <7 cm).
